# The Association Between Distinct Frontal Brain Volumes and Behavioral Symptoms in Mild Cognitive Impairment, Alzheimer's Disease, and Frontotemporal Dementia

**DOI:** 10.3389/fneur.2019.01059

**Published:** 2019-10-03

**Authors:** Antti Cajanus, Eino Solje, Juha Koikkalainen, Jyrki Lötjönen, Noora-Maria Suhonen, Ilona Hallikainen, Ritva Vanninen, Päivi Hartikainen, Matteo de Marco, Annalena Venneri, Hilkka Soininen, Anne M. Remes, Anette Hall

**Affiliations:** ^1^Institute of Clinical Medicine, Neurology, University of Eastern Finland, Kuopio, Finland; ^2^Neurocenter, Neurology, Kuopio University Hospital, Kuopio, Finland; ^3^Combinostics Ltd., Tampere, Finland; ^4^MRC Oulu, Oulu University Hospital, Oulu, Finland; ^5^Department of Radiology, Kuopio University Hospital, Kuopio, Finland; ^6^Department of Neuroscience, University of Sheffield, Sheffield, United Kingdom; ^7^Unit of Clinical Neuroscience, Neurology, University of Oulu, Oulu, Finland

**Keywords:** magnetic resonance imaging, mild cognitive impairment, Alzheimer's disease, frontotemporal dementia, behavioral symptoms

## Abstract

Our aim was to investigate the association between behavioral symptoms of agitation, disinhibition, irritability, elation, and aberrant motor behavior to frontal brain volumes in a cohort with various neurodegenerative diseases. A total of 121 patients with mild cognitive impairment (MCI, *n* = 58), Alzheimer's disease (AD, *n* = 45) and behavioral variant frontotemporal dementia (bvFTD, *n* = 18) were evaluated with a Neuropsychiatric Inventory (NPI). A T1-weighted MRI scan was acquired for each participant and quantified with a multi-atlas segmentation method. The volumetric MRI measures of the frontal lobes were associated with neuropsychiatric symptom scores with a linear model. In the regression model, we included CDR score and TMT B time as covariates to account for cognitive and executive functions. The brain volumes were corrected for age, gender and head size. The total behavioral symptom score of the five symptoms of interest was negatively associated with the volume of the subcallosal area (β = −0.32, *p* = 0.002). High disinhibition scores were associated with reduced volume in the gyrus rectus (β = −0.30, *p* = 0.002), medial frontal cortex (β = −0.30, *p* = 0.002), superior frontal gyrus (β = −0.28, *p* = 0.003), inferior frontal gyrus (β = −0.28, *p* = 0.005) and subcallosal area (β = −0.28, *p* = 0.005). Elation scores were associated with reduced volumes of the medial orbital gyrus (β = −0.30, *p* = 0.002) and inferior frontal gyrus (β = −0.28, *p* = 0.004). Aberrant motor behavior was associated with atrophy of frontal pole (β = −0.29, *p* = 0.005) and the subcallosal area (β = −0.39, *p* < 0.001). No significant associations with frontal brain volumes were found for agitation and irritability. We conclude that the subcallosal area may be common neuroanatomical area for behavioral symptoms in neurodegenerative diseases, and it appears to be independent of disease etiology.

## Introduction

Neurodegenerative diseases exhibit various symptoms affecting patients' behavior and personality. Behavioral symptoms such as agitation, disinhibition, irritability, elation, and aberrant motor behavior have a great effect on patients' social relations, caregiver burden, and quality of life. Some symptoms are more characteristic for specific diseases, but there is overlapping of behavioral symptoms between different diseases. Various behavioral symptoms may be present even in the earliest clinical stages of these diseases and the detection of these symptoms can be valuable for diagnostic purposes. Typical behavioral symptoms in the early stage of Alzheimer's disease (AD) are apathy, agitation, irritability, anxiety, and depression (1–4). Considering bvFTD, typical behavioral symptoms are disinhibition, agitation, apathy, loss of empathy, aberrant motor behavior and preservative compulsive behavior (1, 5, 6). Disinhibition, apathy, emotional bluntness, aberrant motor behavior, stereotypical behaviors and eating abnormalities are more characteristic to bvFTD and have been found to discriminate bvFTD from AD (1, 6–8). Behavioral symptoms are also interrelated with dementia severity in AD. Higher agitation, apathy, disinhibition, irritability, and aberrant motor behavior scores are associated with increased dementia severity in AD (1, 9). In bvFTD, the severity of behavioral symptoms is higher than in AD, yet no difference in severity of behavioral disturbances is observed (10). Behavioral symptoms may also be present in cases with mild cognitive impairment (MCI). Especially symptoms such as apathy, agitation, anxiety, irritability, and depression, are more prevalent in subjects with MCI compared to healthy people (11, 12).

Different neurodegenerative disorders present different pattern of brain atrophy compared to each other. The medial temporal atrophy is typical for AD (13, 14), while anterior temporal and/or frontal lobes, the insula and anterior cingulate cortex are affected in bvFTD (15–19). However, there are variance of the pattern of atrophy within both these diagnostic entities. For instance, bvFTD may present different type of atrophy depending from underlying genetic background (20–24). Even though the patterns of atrophy differ, there are also some overlap of brain areas that are affected in these diseases. Given that also behavioral symptoms overlap in different disease etiologies, it supports the idea that damage in some brain areas or networks are associated with specific types of behavioral changes. Detection of neuroanatomical changes associated with these behavioral changes could improve early diagnostic of dementing diseases. Similar idea has been presented for mental disorders by the Research Domain Criteria (RDoC), suggesting that mental disorders are disorders of brain circuits, and specific behavioral signs and symptoms can be explained with neuroimaging and other brain functional quantification methods *in vivo* (25).

In some studies, the association between a specific behavioral symptom and cortical areas has been identified in AD and bvFTD. Disinhibition has been associated with gray matter atrophy in the orbitofrontal cortex (26, 27). Agitation has been associated with atrophy in the inferior frontal cortex, insula and anterior cingulate cortex (28, 29) and aberrant motor behavior with decreased volume in anterior cingulate gyrus and inferior frontal cortex (27, 28). Similar findings have been also found in other neurodegenerative conditions and also in healthy people (30–32). However, it remains unclear whether these neuroanatomical correlates are disease specific or is there common foci of neural damage associating to behavioral symptoms independently of clinical diagnosis.

The aim of this study was to evaluate common neuroanatomical associations in the frontal area for behavioral symptoms in a cohort of patients with MCI, AD, and bvFTD, irrespective of underlying neurodegenerative disorder. Our hypothesis is, that behavioral symptoms could be driven by common neuroanatomical correlates, regardless of clinical diagnosis. If there were such common associations for behavioral symptoms, this information could be of great value when targeting treatments for individual patients. Contrary to previous studies, our data consist of patients with different neurodegenerative diseases at varying clinical stages allowing us to locate regions of interest in neural networks that associate with behavioral symptoms, regardless of the underlying disease etiology or pathology.

## Materials and Methods

### Participants

We examined a total of 121 subjects including 58 patients with a diagnosis of MCI, 45 AD and 18 bvFTD. The study population consisted of 92 subjects from Kuopio, Finland, and 29 subjects from Sheffield, United Kingdom (Table 1). The data are part of the VPH-DARE@IT project. The diagnoses of neurodegenerative diseases were formulated by an experienced neurologist specialized in memory disorders according to standard clinical criteria (16, 33–36). We included only patients with an appropriate T1-weighted MRI scan who had a correctly completed Neuropsychiatric Inventory (NPI). Mini-Mental State Examination (MMSE) scores, Clinical Demenita Rating (CDR) scores and Trail Making B test time were also determined.

**Table 1:** Demographic and clinical data according to clinical diagnosis.

	**Total**	**MCI**	**AD**	**bvFTD**	***p***
*N* (%)	121 (100%)	58 (48%)	45 (37%)	18 (15%)	
Gender, female, % *(N)*	53% (64)	57% (33)	47% (21)	57% (10)	0.6
Age, mean (SD)	68.2 (9.4)	69.4 (9.0)	68.8 (9.6)	62.6 (8.5)	0.02
Education years, mean (SD)	11.3 (3.5)	11.4 (3.7)	11.4 (3.6)	10.6 (2.6)	0.7
MMSE score, mean (SD)	23.5 (4.8)	26.3 (2.5)	21.5 (4.9)	19.8 (5.4)	<0.001
CDR score, mean (SD)	0.7 (0.5)	0.48 (0.1)	1.0 (0.6)	0.8 (0.6)	<0.001
TMT B time, sec, mean (SD)	242 (159)	194 (99)	311 (215)	240 (83)	0.001
Total NPI score, mean (SD)	8.8 (12.5)	7.2 (11.4)	9.4 (12.5)	12.7 (15.5)	0.3

*Statistical significance between groups was assessed with ANOVA and chi square test*.

The ethics committee of the Northern Savonia Hospital District for the Kuopio cohort and the Yorkshire and Humber Regional Ethics Committee (Ref No: 12/YH/0474) for the Sheffield cohort approved the research protocol in accordance with the principles of the Declaration of Helsinki. Written informed consent was obtained from all participants prior to enrollment.

All included patients were 50–85 years old. Inclusion criteria for the MCI patients were set as follows: MCI diagnosis as evidenced by the following: referral because of cognitive impairments and diagnosis of amnestic or non-amnestic MCI (33, 34). AD patients were included if they met the diagnostic criteria for probable AD according to NINCDS-ADRDA Alzheimer's criteria (36) or the diagnosis of prodromal AD according to Dubois et al. (35). bvFTD patients were included if they met the Neary et al. clinical diagnostic criteria for FTD (16). The recruiting of study population was initiated before the establishment of the international consensus criteria for behavior variant frontotemporal dementia (15), thus the consensus criteria by Neary and colleagues were used in the recruitment. However, we retrospectively evaluated the bvFTD patient's data and all subjects fulfilled the criteria for at least clinical probable bvFTD, presenting significant functional decline and positive imaging findings (15).

Patients were excluded if they had obvious brain, systemic or psychiatric disorders that could possibly affect cognitive functions such as stroke, severe depression or endocrine disorders. Also, MCI patients were excluded if they met the diagnostic criteria of dementia according to DSM-IV at baseline.

### Neuropsychiatric Evaluation

**Figure 1:**
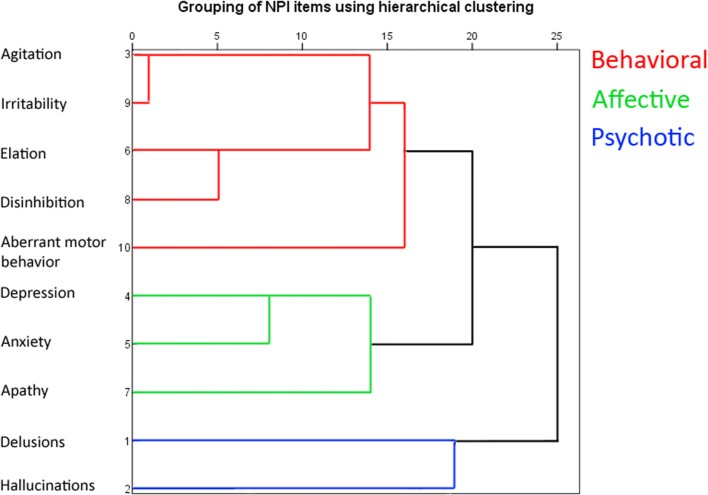
Hierarchical clustering of the NPI symptoms shown as a dendrogram. The clustering was done with between-groups linkage using Pearson's correlation as the distance between the NPI items.

Behavioral symptoms were evaluated using the NPI (37). The NPI is a structured interview administered to patients' caregivers. It consists of 10 or 12 separate items assessing neuropsychiatric disturbances common in dementia, including delusions, hallucinations, agitation/aggression, depression/dysphoria, anxiety, elation/euphoria, apathy/indifference, disinhibition, irritability/lability, aberrant motor behavior, nighttime behaviors and appetite/eating disorders. In this study, we used the original 10 items and further grouped those symptoms into three categories, behavioral, affective and psychotic symptoms according to a hierarchical clustering of the NPI items (Figure 1). The clustering of our cohort was similar to the results from a factorial analysis performed with a larger dementia cohort (38). We performed a preliminary analysis for the neuroanatomical correlates with all neuropsychiatric clusters. This analysis revealed significant correlations with frontal lobe structures only with behavioral symptoms, thus we focused our more specific analyses only on behavioral symptoms and excluded affective and psychotic items (results of correlation analysis for affective and psychotic symptoms in Supplementary Figure 1). Eating abnormalities and nighttime behaviors were also excluded from analyses, since they are not part of the core NPI questionnaire, and these items include several types or positive and negative symptoms (e.g., hyperorality vs. lack of appetite). The clustering resulted in behavioral symptoms including agitation, irritability, disinhibition, elation, and aberrant motor behavior. The frequency and severity of each item were rated. Composite scores for each individual neuropsychiatric symptom were calculated by multiplying the frequency score (1–4, occasionally-very often) by severity score (1–3, mild-difficult), making 12 the highest possible score for each item. A behavioral symptoms total score was calculated by adding up the composite scores of agitation, irritability, disinhibition, elation and aberrant motor behavior, making the maximum score 5*12 = 60.

### MRI Analysis

MRI scans were acquired on a Phillips Achieva 3T scanner in Kuopio and Phillips Ingenia 3T scanner in Sheffield using a shared image acquisition protocol. Gradient echo 3D T1-weighted images were acquired sagittally with the following parameters: voxel size: 0.94 × 0.94 × 1.00 mm; repetition time: 8.2 ms; echo delay time: 3.8 ms; field of view: 256 mm; matrix size: 256 × 256 × 170.

Image segmentation was performed using the commercial cNeuro® cMRI image quantification tool (Combinostics, Tampere, Finland). The tool segments images to 133 areas by using a multi-atlas segmentation framework (39). The method consists of the following steps: (1) atlas selection is performed, (2) a probabilistic atlas is generated by registering non-rigidly the selected atlases to the patient image and (3) expectation maximization classification is applied for producing the final segmentation (39). The volumes were normalized first for head size (40) and then for age and gender (41).

We focused our analyses to the frontal lobes and excluded other areas in order to maintain sufficient statistical power and due to findings of existing literature. A total of 11 different brain areas of the frontal lobe was selected and the total volumes of the frontal lobes to be used in the analyses included anterior, posterior, lateral and medial orbital gyrus, gyrus rectus, medial frontal cortex, frontal pole, superior frontal gyrus, middle frontal gyrus, inferior frontal gyrus, and the subcallosal area. The volume of the inferior frontal gyrus was calculated by adding three separate segments together: opercular, triangular, and orbital part of the inferior frontal gyrus. The rest of the areas were analyzed as they were segmented by the multi-atlas segmentation method. Any brain areas outside the frontal lobes were excluded.

### Statistical Analysis

The NPI items were grouped with hierarchical clustering using between-groups linkage and Pearson's correlation as the distance between items. The demographic and clinical data between diagnostic groups were analyzed with one-way ANOVA and chi-square tests. The initial correlation analysis of all segmented brain volumes and neuropsychiatric symptoms was done with two-tailed Pearson's correlation. Further analyses of the association between behavioral symptom scores and frontal brain volumes was tested with a linear regression model using CDR (Clinical Dementia Rating) score and TMT-B (Trail-Making Test, B) time as confounders, to control the effect of subjects' cognitive state and executive functioning. We also examined differences in volumetric brain measures in different groups with one-way ANOVA, using Tukey's *post hoc* analysis. We chose to only discuss results with *p* ≤ 0.005 as significant due to multiple testing. The analyses were performed with IBM SPSS Statistics, version 25.

## Results

The bvFTD group was on average 6 years younger compared to the AD group and 7 years younger than the MCI group. The MMSE score was higher in the MCI group compared to the AD and bvFTD groups. There were no significant differences in the degree of education, gender distribution or total NPI scores among the three groups. The demographic data are presented in Table 1.

The frequency of behavioral symptoms and the composite NPI scores according to clinical diagnoses are shown in Table 2. The composite scores (frequency^*^severity) in all studied behavioral symptom categories were higher in patients with bvFTD. Disinhibition scores were significantly higher in the bvFTD group compared to the AD and MCI groups. Aberrant motor behavior and elation scores were also higher in bvFTD compared to MCI. Irritability was the most frequent symptom in the whole group (33%) as well as in patients with AD (38%) and MCI (31%). Disinhibition and aberrant motor behavior were most common in patients with bvFTD (33 and 28%, respectively). However, there were no statistical differences in the frequency of any of the behavioral symptoms among the three groups.

**Table 2:** Total scores and frequency of NPI behavioral symptoms according to clinical diagnosis.

**Diagnosis**		**Behavioral symptoms total**	**Agitation**	**Elation**	**Disinhibition**	**Irritability**	**Aberrant motor behavior**
MCI	Score mean (SD)	1.7 (3.2)^*^	0.4 (1.1)	0.1 (0.4)^*^	0.2 (0.4)^*^	0.8 (1.4)	0.3 (1.2)^*^
	Frequency	41 %	17 %	9 %	14 %	31 %	10 %
AD	Score mean (SD)	3.2 (5.5)	0.8 (1.7)	0.4 (1.2)	0.4 (1.2)^*^	1.1 (2.0)	0.6 (1.4)
	Frequency	53 %	31 %	11 %	18 %	38 %	20 %
bvFTD	Score mean (SD)	6.3 (10.7)^*^	1.1 (2.2)	0.9 (2.9)^*^	1.4 (2.6)^*^	1.2 (2.6)	1.7 (2.9)^*^
	Frequency	44 %	28 %	17 %	33 %	28 %	28 %
Total	Score mean (SD)	3.0 (5.9)	0.6 (1.5)	0.3 (1.4)	0.4 (1.3)	1.0 (1.8)	0.6 (1.7)
	Frequency	46 %	24 %	11 %	18 %	33 %	17 %
	*P*-value	0.01	0.18	0.08	<0.01	0.51	0.01

*Frequency was calculated as a dichotomous variable, based on whether the patient did have symptoms of any severity or did not, and is presented as percentage of the population*.

The mean values of symptom total scores (frequency*severity) are presented separately for each clinical diagnosis group and for the whole cohort. Statistical significance of symptom scores were estimated with one-way ANOVA using Tukey's post hoc analysis.

* *Statistically significant difference (p ≤ 0.05) in between MCI/AD and bvFTD*.

**Figure 2:**
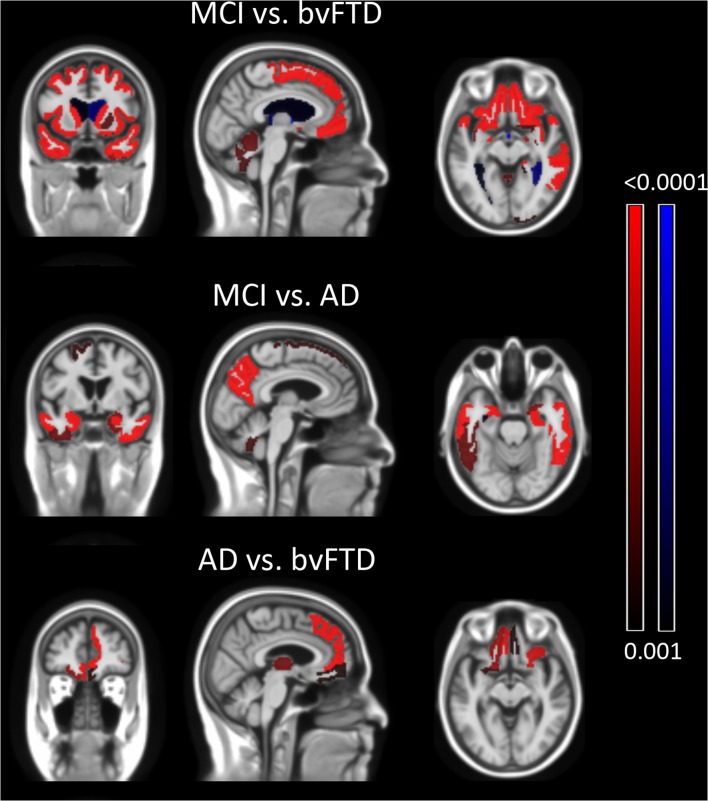
Group comparison of brain structure volumes of different diagnostic groups. Top row illustrates the comparison between bvFTD and MCI groups. The bvFTD group shows frontal and temporal atrophy and also reduced volume of deep gray matter structures. The middle row shows AD group's temporal and parietal atrophy compared to the MCI group. The lowest row shows the bvFTD group having reduced orbitofrontal, medial frontal and thalamic volumes compared to AD. The AD group showed no volume reduction compared to the bvFTD group. Comparison was done with two-tailed *t*-test and only significant areas of *p* ≤ 0.001 are highlighted.

One-way ANOVA and Tukey's *post hoc* analysis showed that the volume of the subcallosal area did not differ among the three diagnostic groups. All other frontal lobe volumes showed statistically significant differences among the three groups (Table 3). The comparison of brain volumes between different diagnostic groups is shown in Figure 2. Compared to MCI, the bvFTD group showed extensive atrophy in frontal cortex, striatum and temporal lobes, and also enlarged lateral ventricles. Marked parietal and also temporal atrophy was detected in AD compared to MCI cases. The comparison between AD and bvFTD revealed prominent medial frontal and orbitofrontal atrophy in bvFTD group, while none of evaluated brain areas was smaller in the AD group compared to bvFTD.

**Table 3:** Comparison of brain volumes of the frontal lobes based on clinical diagnoses.

**Combined volumes of R&L hemispheres, cm^**3**^**	**Total**	**MCI**	**AD**	**bvFTD**	***p*-value, all groups**	***p*-value, AD vs. bvFTD**
Anterior orbital gyrus, mean (SD)	3.4 (0.6)	3.6 (0.6)	3.4 (0.6)	3.0 (0.6)	0.001	0.020
Posterior orbital gyrus, mean (SD)	5.0 (0.7)	5.2 (0.6)	5.0 (0.6)	4.3 (0.8)	<0.001	<0.001
Lateral orbital gyrus, mean (SD)	3.1 (0.6)	3.4 (0.5)	3.0 (0.5)	2.6 (0.6)	<0.001	0.006
Medial orbital gyrus, mean (SD)	7.3 (0.9)	7.6 (0.6)	7.4 (0.8)	6.4 (1.1)	<0.001	<0.001
Gyrus rectus, mean (SD)	2.9 (0.5)	3.0 (0.4)	2.9 (0.4)	2.4 (0.5)	<0.001	<0.001
Medial frontal cortex, mean (SD)	2.5 (0.5)	2.7 (0.4)	2.5 (0.5)	1.9 (0.5)	<0.001	<0.001
Frontal pole, mean (SD)	6.0 (0.8)	6.2 (0.6)	6.0 (0.7)	5.3 (1.1)	<0.001	0.008
Superior frontal gyrus, mean (SD)	24.8 (2.8)	25.9 (2.2)	24.3 (2.2)	22.5 (3.7)	<0.001	0.030
Middle frontal gyrus, mean (SD)	31.8 (4.0)	33.2 (2.7)	31.4 (4.0)	28.1 (5.2)	<0.001	0.004
Inferior frontal gyrus, mean (SD)	13.1 (1.7)	13.7 (1.6)	13.0 (1.4)	11.5 (2.0)	<0.001	0.002
Subcallosal area, mean (SD)	2.6 (0.4)	2.6 (0.3)	2.6 (0.4)	2.4 (0.4)	0.145	0.160
Frontal lobe, mean (SD)	184 (17)	191 (12)	183 (15)	163 (21)	<0.001	<0.001

*The values represent combined right and left hemisphere volumes mean (SD) values of specific areas (cm^3^). The volumes were corrected for age, sex and head size. All frontal areas except the subcallosal area (p = 0.145) showed a statistically significant difference between groups. Statistical significance was assessed with one-way ANOVA in addition with Tukey's post hoc analysis*.

**Figure 3:**
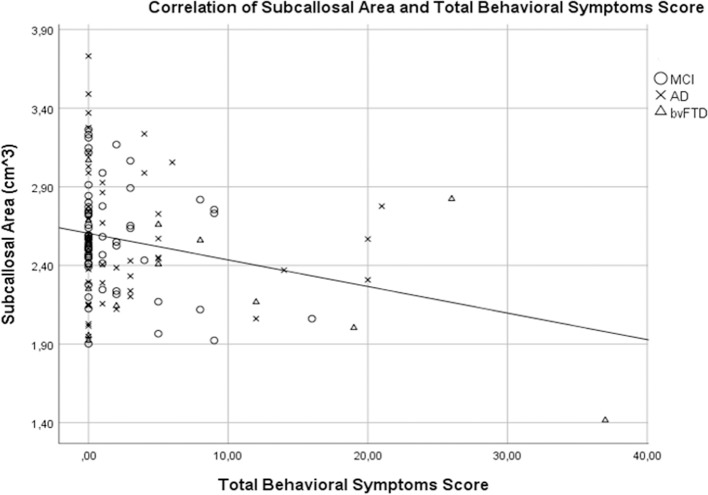
Association of the volume of the subcallosal area and total behavioral scores. A regression analysis with CDR score and TMT-B time as a confounders revealed a significant association between the volume of the subcallosal area and the total behavioral score (β = −0.32, *p* = 0.002).

**Figure 4:**
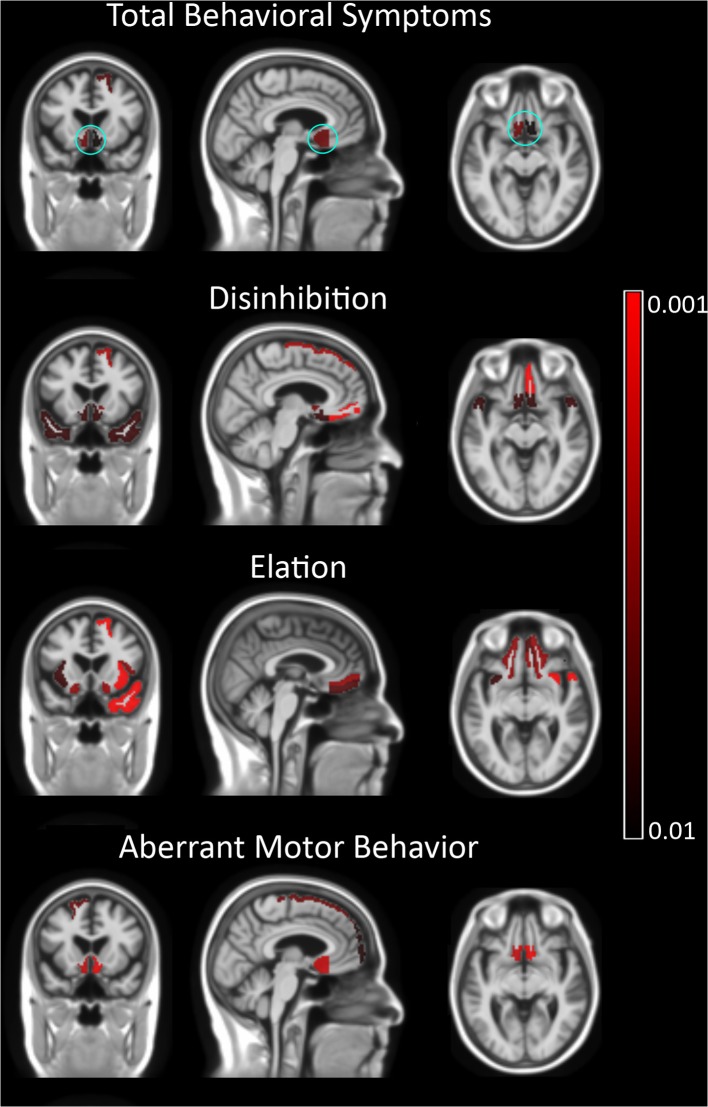
Correlation of the total and individual behavioral symptoms with brain volumes. In the first row, total behavioral symptom scores show a correlation with the subcallosal area and left superior frontal gyrus. All three symptom scores show a correlation with ventromedial prefrontal cortex. The left superior frontal gyrus showed a correlation with elation and disinhibition, while aberrant motor behavior was associated with right superior frontal gyrus. In addition to frontal volumes, disinhibition scores were associated with the bilateral temporal pole. The subcallosal area is shown in cyan circle in the top row. Irritability and agitation showed no correlation with any focal brain volume. Negative correlations are shown in red. No positive correlations were detected.

We analyzed the association between NPI behavioral symptom total scores and frontal volumes in all patients using the CDR score and TMT-B time confounders (Table 4). There was significant association between the total behavioral symptom score of these five symptoms and the volume of the subcallosal area (β = − 0.32, *p* = 0.002) (Figure 3). Disinhibition scores were associated with reduced volumes in the gyrus rectus (β = − 0.30, *p* = 0.002), medial frontal cortex (β = − 0.30, *p* = 0.002), superior frontal gyrus (β = −0.28, *p* = 0.004), inferior frontal gyrus (β = −0.28, *p* = 0.005) and in the subcallosal area (β = − 0.28, *p* = 0.005). Aberrant motor behavior scores were associated with reduced volume of frontal pole (β = −0.29, *p* = 0.005) the subcallosal area (β = − 0.39, *p* < 0.001). Elation was associated with the medial orbital gyrus (β = −0.30, *p* = 0.002) and inferior frontal gyrus (β = −0.28, *p* = 0.004). There was no association between agitation and irritability with any focal or gross frontal volumes. Visualizations of the correlations between behavioral symptoms and brain areas are shown in Figure 4.

**Table 4:** Standardized regression coefficients (β) from the linear regression model of frontal volumes and behavioral symptoms.

	**Behavioral symptoms total** **β**	**Agitation** **β**	**Disinhibition** **β**	**Elation** **β**	**Irritability** **β**	**Aberrant motor behavior** **β**
Anterior orbital gyrus	0.091	0.133	0.072	−0.040	0.160	−0.005
Posterior orbital gyrus	−0.089	−0.015	−0.167	−0.146	0.087	−0.150
Lateral orbital gyrus	−0.061	0.063	−0.235	−0.206	0.155	−0.025
Medial orbital gyrus	−0.204	0.043	−0.257^*^	−0.302^**^	−0.025	−0.186
Gyrus rectus	−0.193	−0.092	−0.301^**^	−0.230	−0.024	−0.124
Medial frontal cortex	−0.164	−0.031	−0.302^**^	−0.248^*^	0.020	−0.107
Frontal pole	−0.194	0.089	−0.171	−0.233	−0.044	−0.292^**^
Superior frontal gyrus	−0.222	−0.060	−0.277^**^	−0.237	−0.089	−0.205
Middle frontal gyrus	−0.009	0.098	−0.123	−0.101	0.188	−0.164
Inferior frontal gyrus	−0.214	−0.120	−0.276^**^	−0.279^**^	−0.073	−0.088
Subcallosal area	−0.317^**^	−0.140	−0.282^**^	−0.262^*^	−0.115	−0.387^**^
Frontal lobe	−0.139	0.014	−0.265^*^	−0.233^*^	0.064	−0.163

*The behavioral symptoms column represents the total score of the five individual behavioral symptoms (agitation, disinhibition, elation, irritability and aberrant motor behavior) and combined score of these five symptoms. The models are corrected for CDR score and TMT-B time. The volumes have been pre-corrected for age, gender and head size. Results with statistical significance of p ≤ 0.005 are in bold*.

* *p ≤ 0.01*;

** *p ≤ 0.005*.

## Discussion

We evaluated the behavioral and frontal volume associations in a cohort of patients at several clinical stages and with different etiologies of neurodegenerative diseases. As expected, frontal lobe volumes were the most preserved in MCI and the most reduced in bvFTD. Of separate frontal volumes, smaller volumes in the subcallosal area were associated with higher disinhibition and aberrant motor behavior scores, as well as the total behavioral symptoms score. Higher elation scores also showed a suggestive association with lower subcallosal volume, however not reaching statistical significance. There was no difference in the volume of the subcallosal area among the three groups, emphasizing its correlations to behavioral symptoms being independent of underlying disease etiology and stage of cognitive impairment (Table 3).

In addition to the subcallosal area, higher disinhibition scores were associated with decreased volume of ventromedial and lateral prefrontal structures, more specifically the gyrus rectus, medial frontal cortex and superior and inferior frontal gyrus. Decreased volumes of these areas have also been associated with disinhibited behavior in bvFTD and AD patients (27, 28, 30, 32, 42). It has also been postulated that both disinhibition and apathy associate with orbitofrontal and medial frontal cortices (43). However, analyses did not show any association between apathy and analyzed frontal lobe volumes (Supplementary Figure 1). Taken together previous and our findings it can be assumed that the symptoms of aberrant motor behavior and disinhibition are strongly associated with damage to the subcallosal area, the end-point of the uncinate fasciculus.

To our knowledge there is no previous work assessing the neural correlates of elation/euphoria in neurodegenerative diseases. However, there is one study evaluating association between sensitivity to drug reward (measured as euphoria) and functional brain correlates in healthy people. This study suggested reduced activity in the middle frontal gyrus and poor inhibitory control to be associated with greater drug reward experienced as euphoria (44). Our data showed an association between elation and inferior frontal gyrus and medial orbital gyrus. We suggest that the dysfunction/atrophy of lateral prefrontal cortex might be associated with elation with coexisting poor inhibition in patients with dementia.

The subcallosal area is a small area located in the medial frontal cortex below the genu of the corpus callosum and anterior cingulate gyrus, caudal to the gyrus rectus. This area is located in the path of the uncinate fasciculus, a bilateral white matter tract connecting the orbitofrontal cortex and the temporal lobe. The fibers of the uncinate fasciculus connect the amygdala and the uncus to the subcallosal area (45). Decreased volumes in the subcallosal area have been previously associated with disinhibition (27, 42). Also, reduced integrity of the uncinate fasciculus has been detected in bvFTD, AD and psychiatric disorders and the reduction of integrity of this specific tract has been found to associate with disinhibition (26, 42, 46, 47) and aberrant motor behavior (28). Our results support previous findings and also suggest that damage to this specific area associates with frequency and severity of disinhibition and aberrant motor behavior, regardless of the underlying etiology of neurodegeneration. This information could be of great value, considering possible symptom specific treatments such as transcranial magnetic stimulation (TMS), similarly as used in psychiatric conditions for therapeutic purposes. Yet, more studies with larger cohorts and with different diseases are needed to validate these results.

### Strengths and Weaknesses

The patients were clinically well-examined by a neurologist specialized in memory disorders and diagnosed according to recent clinical guidelines for MCI, AD, and bvFTD. As limitations, we did not have healthy controls with a neuropsychiatric evaluation, thus the study population consisted only of subjects with subjective and/or evident clinical symptoms. By contrary, this is the first study where neuropsychiatric symptoms and neuroanatomical correlations has been evaluated with MCI cases with very mild cognitive symptoms. Our study setting was cross-sectional, meaning variability in frequency and severity of neuropsychiatric symptoms and brain volumes between diseases represents the differences only at the time of clinical diagnosis. In future studies we emphasize the importance of longitudinal assessment of atrophy rate and behavioral symptoms. We also performed a similar regression analysis with different diagnostic groups separately and the associations (β-values) between neuropsychiatric symptoms and frontal volumes were similar to those performed with the whole cohort. However, due to the small sample sizes of each diagnostic group, our analyses were unpowered, and the results did not reach statistical significance. Also, we needed to focus on frontal lobes, since due to small sample size whole brain analyses would have been unpowered. The MRI scans were obtained with a homogenous protocol and segmented with a reliable and validated multi-atlas method. The scans were performed with different scanners, however the segmentation method has been shown to be relatively robust over different scanner models. The results did not survive correction for multiple testing (Bonferroni correction). However, we set the threshold for statistical significance at *p* ≤ 0.005 for associations of frontal volumes and behavioral symptoms and *p* ≤ 0.001 for differences in frontal brain volumes between groups in order to maintain reliability of the results.

## Conclusions

Our findings show that behavioral symptoms in neurodegenerative dementing diseases are associated with atrophy of specific frontal cortical areas and associations appear to be independent of disease etiology and cognitive performance. Especially, the subcallosal area was found to associate well with the behavioral symptoms regardless of clinical diagnosis, thus damage to this area may be a common neuroanatomical area for behavioral symptoms in neurodegenerative diseases independent of the specific type of dementia.

## Data Availability Statement

The datasets for this manuscript are not publicly available because of data confidentiality. Our data concerns delicate patient information and our study consent does not allow us to share the data publicly.

## Ethics Statement

The ethics committee of the Northern Savonia Hospital District for the Kuopio cohort and the Yorkshire and Humber Regional Ethics Committee (Ref No: 12/YH/0474) for the Sheffield cohort approved the research protocol in accordance with the principles of the Declaration of Helsinki. Written informed consent was obtained from all participants prior to enrollment.

## Author Contributions

AC, AH, and AR contributed conception and design of the study. AC wrote the first draft of the manuscript. All authors contributed to manuscript revision, read and approved the submitted version.

### Conflict of Interest

JK and JL are shareholders and founders of Combinostics Ltd. The remaining authors declare that the research was conducted in the absence of any commercial or financial relationships that could be construed as a potential conflict of interest.

## Abbreviations

AD: Alzheimer's Disease
bvFTD: Behavioral variant frontotemporal dementia
MCI: Mild cognitive impairment
NPI: Neuropsychiatric inventory.
