# Supplementary figures and images for: The Association Between Distinct Frontal Brain Volumes and Behavioral Symptoms in Mild Cognitive Impairment, Alzheimer's Disease, and Frontotemporal Dementia
Source: Front Neurol. 2019 Oct 3;10:1059. doi: 10.3389/fneur.2019.01059 (PMC6786130; doi:10.3389/fneur.2019.01059)

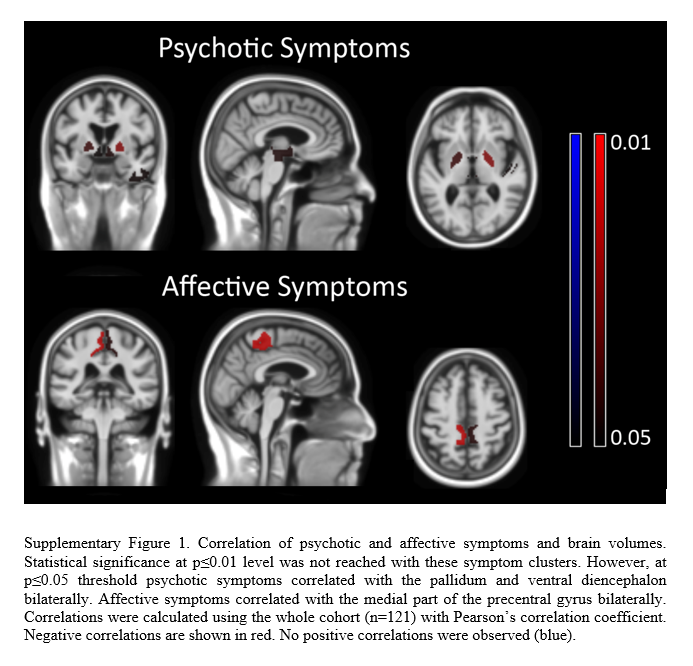

Supplement: Supplementary file 1 [file Image_1.tif]
